# Antimicrobial Susceptibility Profile of Bacterial Isolates Among Patients Admitted to the Neurosurgery Intensive Care Unit of a Tertiary Care Hospital: A Cross-Sectional Study

**DOI:** 10.7759/cureus.93819

**Published:** 2025-10-04

**Authors:** Smrutisree Mohapatra, Nipa Singh, Jyoti Prakash Sahoo, Narendra Kumar Das, Basanti Kumari Pathi

**Affiliations:** 1 Microbiology, Kalinga Institute of Medical Sciences, Bhubaneswar, IND; 2 Pharmacology, Kalinga Institute of Medical Sciences, Bhubaneswar, IND; 3 Neurological Surgery, Kalinga Institute of Medical Sciences, Bhubaneswar, IND

**Keywords:** antibiotic therapy, antimicrobial susceptibility testing, bacterial drug resistance, culture and sensitivity, enterobacterales, gram-positive cocci, healthcare-associated infection, hospital-acquired infection, non-fermenters, traumatic brain injury (tbi)

## Abstract

Background and objectives: Patients admitted to the neurosurgery intensive care unit (ICU) are often exposed to mechanical ventilation, invasive procedures, and polypharmacy. Prolonged hospital stays and multiple antibiotics facilitate the development of hospital-acquired infections (HAI). We planned this study to determine the bacteriological profile and antimicrobial susceptibility testing (AST) patterns among neurosurgery ICU patients.

Methods: This cross-sectional study was conducted from May 2024 to April 2025 at the Kalinga Institute of Medical Sciences (KIMS) in Bhubaneswar, India. This study included adult patients hospitalized in the neurosurgery ICU within the above-mentioned time frame. Specimens like endotracheal tube (ETT) aspirate, blood, urine, and body fluids were collected from the eligible participants and were assessed for the bacteriological profile. Culture media like MacConkey agar, chocolate agar, 5% sheep blood agar, and cysteine lactose electrolyte-deficient (CLED) agar were employed for the growth of pathogenic bacteria. For AST, we used the VITEK 2 system (bioMérieux, Marcy-l'Étoile, France). We used the R software Version 4.4.3 (R Foundation for Statistical Computing, Vienna, Austria) for analysis.

Results: Four hundred twenty-five patients were eligible for the study. The male-to-female ratio was 3:2. The average age of the participants was 58.9 (51.7-72.3) years. The majority of samples were obtained from ETT (308, 72%), followed by blood (79, 19%) and others like urine, pus, etc. (38, 9%), respectively. Most of the pathogens belonged to *Enterobacterales* (253, 59.5%), followed by non-fermenters (130, 30.6%) and Gram-positive cocci (42, 9.9%), respectively. *Klebsiella pneumoniae* (162, 38.1%), *Acinetobacter baumannii* (71, 16.7%), *Pseudomonas aeruginosa* (50, 11.8%), *Escherichia coli* (46, 10.8%), and *Staphylococcus aureus* (32, 7.5%) were the most common pathogenic bacteria. We also discovered some emergent pathogens like *Proteus mirabilis* (21, 4.9%), *Serratia marcescens* (7, 1.6%), *Elizabethkingia meningoseptica* (2, 0.5%), and *Morganella morganii* (1, 0.2%). *Klebsiella pneumoniae*, *Escherichia coli*, and *Acinetobacter baumannii* isolates demonstrated the highest sensitivity towards tigecycline. *Pseudomonas aeruginosa* isolates were highly sensitive to cefoperazone-sulbactam and imipenem. Antibiotics like linezolid, vancomycin, tigecycline, daptomycin, and teicoplanin were very effective against* Staphylococcus aureus*.

Conclusion: ETT was the most common sample collected, and most pathogens were from *Enterobacterales*.The most prevalent pathogens were *Klebsiella pneumoniae*, *Acinetobacter baumannii*, *Pseudomonas aeruginosa*, *Escherichia coli*, and *Staphylococcus aureus*. We also noticed a few cases of emerging pathogens like *Elizabethkingia meningoseptica* and *Morganella morganii*. *Acinetobacter baumannii*, *Escherichia coli*, and *Klebsiella pneumoniae* were highly sensitive to tigecycline. Cefoperazone-sulbactam was highly effective against *Pseudomonas aeruginosa*. *Staphylococcus aureus* isolates showed maximum sensitivity towards linezolid and vancomycin.

## Introduction

Healthcare-associated infections (HCAIs) are more prevalent among neurosurgical patients than other critically ill patients [1,2]. Despite the relatively low incidence of postoperative central nervous system infections (PCNSIs), patients frequently have poor outcomes due to major complications after neurosurgery, particularly craniotomies [3,4]. Meningitis, subdural empyema, epidural abscess, and brain abscess are the most typical manifestations of PCNSI [3]. Mortality rate increases by four times among patients with traumatic brain injury (TBI) after developing pneumonia [5]. Sepsis and septicemia increase the risk of mortality with a hazard ratio (HR) of 4.97 [6].

Many factors influence the emergence of infection in these instances. Long-term antibiotic usage, mechanical ventilation, indwelling invasive devices, aspiration of gastric content, weakened host immunity, and a Glasgow Coma Scale (GCS) score of <8 are all acknowledged as risk factors for infections in ICU settings [7-9]. Peripheral immune cells enter the brain parenchyma in response to acute brain injury, aggravating neuroinflammation and neurovascular damage [10,11]. By producing reactive nitrogen and oxygen species, as well as inflammatory cytokines, neutrophils cause brain injury [10,12]. The hallmarks of infection susceptibility and the peripheral cellular immune response suppression include monocyte deactivation and temporary lymphopenia [13,14].

Infection source, age, underlying disease, geographical distribution, and irrational antibiotic use all affect the epidemiology of bacterial infections [15,16]. *Pseudomonas aeruginosa*, *Escherichia coli*, *Staphylococcus aureus*, *Acinetobacter baumannii*, and *Klebsiella pneumoniae* are the most frequent pathogens that cause bacterial infections in ICU patients [17-19]. *Serratia marcescens*, *Elizabethkingia meningoseptica*, *Proteus mirabilis*, and *Enterococcus* species have all been responsible for increased infections recently [20,21].

The endotracheal tube (ETT) aspirate, blood, and other specimens (such as urine, pus, cerebrospinal fluid, skin, body fluid, and nasal swabs) are well-known for identifying pathogens in ICU patients [22]. Frequent antimicrobial susceptibility testing (AST) and antibiotic prophylaxis alter the drug susceptibility patterns of pathogenic microorganisms [19,23]. We carried out this study to ascertain the bacteriological profile of patients admitted to the neurosurgery ICU and their AST patterns.

## Materials and methods

Between May 2024 and April 2025, the study was conducted at the Kalinga Institute of Medical Sciences (KIMS) in Bhubaneswar, India. In order to determine the bacteriological profile and their AST patterns, we analyzed the culture findings of patients admitted to the neurosurgery ICU in this cross-sectional study. Before commencing the study, we obtained ethical approval from the Institutional Ethics Committee of the Kalinga Institute of Medical Sciences (KIMS) (approval number: KIIT/KIMS/IEC/1796/2024; date: 24.04.2024). The study complied with the Declaration of Helsinki, institutional norms, and Good Laboratory Practices.

Study criteria

This study included adult patients hospitalized in the neurosurgery ICU within the above-mentioned time frame who acquired an infection after 48 hours of their ICU admission. We excluded patients under 18, those with an infection during ICU admission, or those referred from another hospital with ongoing antibiotics. Additionally, we excluded clinical samples unfit for processing in the microbiology laboratory because of any deficiencies in the pre-analytical stage and patients who passed away before the sample collection for AST.

Study procedure

Sample Collection and Processing

Clinical specimens such as blood, urine, body fluids, and respiratory (from ETT) samples were obtained and sent immediately to the microbiology laboratory. Gram staining was performed on respiratory samples and body fluids, after which they were inoculated into blood agar and MacConkey agar and left overnight in an incubator with 5% CO₂. The blood culture vials were put into the BacT/Alert 3D (bioMérieux, Marcy-l'Étoile, France), a fully automated identification device for bacterial growth detection. After the bottle tested positive, Gram staining was done. Then the bacteria were isolated by aerobic incubation at 37°C for 18-24 hours. This was followed by sub-culturing on 5% sheep blood agar, chocolate agar, and MacConkey agar plates. After inoculating urine samples into cysteine lactose electrolyte-deficient (CLED) agar, the bacteria were isolated using an aerobic overnight incubation at 37°C.

VITEK 2 System (bioMérieux, Marcy-l'Étoile, France)

Initial identification was done using enzymatic tests such as oxidase, coagulase, and catalase. Antimicrobial susceptibility was evaluated, and isolates were identified using the VITEK 2 system, which used the Clinical and Laboratory Standards Institute (CLSI) 2022 cut-off values [24]. The colorimetric reagent card deployed in the VITEK 2 system had 64 wells. Gram-positive and Gram-negative bacteria were determined by utilizing different cards. The organisms in the well were identified by their metabolic activities. The organism was identified with high confidence by comparing its response pattern to a database.

AST

It works by dilution of the micro-broth. In the card wells, the antimicrobial agents were diluted twice. The well was injected with the organism suspension. The maximum dilution of an antimicrobial treatment that prevented the organism's growth was known as the minimum inhibitory concentration (MIC). Using saline solution or broth, colonies were formed after being separated from an agar plate for 18-24 hours. Suspension turbidity was controlled to 0.5 McFarland using a calibrated photometric device (Densichek, bioMérieux, Marcy-l'Étoile, France). A filling tube automatically injected the AST inoculum onto a 64-well colorimetric reagent card with a predetermined antibiotic concentration. Optical readings were taken every 15 minutes while the cards were incubated to evaluate light transmission across the wells, including the growth control well. Each well's MIC data and growth kinetics were interpreted using validated software. The Advanced Expert System (AES) confirmed the final MIC result before providing the antimicrobial susceptibility.

Statistical analysis

Convenience sampling was employed in this cross-sectional study. The data distribution's normality was verified using the Shapiro-Wilk test. For the categorical data, summary statistics were the number and percentage. For the continuous data, summary statistics were the median and the interquartile range (IQR). The study population's distribution was depicted using a mosaic plot. We divided the participants into two groups: adults (≤65 years) and seniors (>65 years). Using chord diagrams, we illustrated the AST results of pathogenic bacteria (i.e., resistant, intermediate, or sensitive). The R software Version 4.4.3 (R Foundation for Statistical Computing, Vienna, Austria) was used to compute the data [25]. A p-value was deemed statistically significant if it was 0.05 or less.

## Results

During the study period, 1589 patients were hospitalized in the neurosurgery ICU. One hundred forty-one (8.9%) of them were younger than 18 years. Referrals for 467 (29.4%) patients with ongoing antibiotics came from other hospitals. At the time of ICU admission, 76 patients (4.8%) had pre-existing infections. During the pre-analytic phase, 289 samples (18.2%) were deemed unsuitable for AST. Within 48 hours after an ICU admission, 191 patients (12%) passed away. Analysis was done on the bacteriological profile of the remaining 425 (26.7%) patients. The study participants' demographic and microbiological characteristics are displayed in Table 1. Of the participants, 170 (40%) were female. The research population's median age was 58.9 (51.7-72.3) years. The median GCS score at admission was 7.0 (4.0-9.0). The *Enterobacterales* most frequently observed were *Klebsiella pneumoniae* (162, 38.1%) and *Escherichia coli* (46, 10.8%). Additional *Enterobacterales* were *Proteus mirabilis* (21, 4.9%), *Enterobacter cloacae *complex (12, 2.8%), *Serratia marcescens* (7, 1.6%), *Providencia stuartii* (2, 0.5%), *Salmonella typhi* (2, 0.5%), and *Morganella morganii* (1, 0.2%). In our investigation, we found the following non-fermenters: *Acinetobacter baumannii* (71, 16.7%), *Pseudomonas aeruginosa* (50, 11.8%), *Burkholderia cepacia* (7, 1.6%), and *Elizabethkingia meningoseptica* (2, 0.5%). The following Gram-positive cocci were found in our study population: *Staphylococcus aureus* (32, 7.5%), *Enterococcus faecalis* (8, 1.9%), and *Enterococcus faecium* (2, 0.5%).

**Table 1:** Demographic and microbiological parameters of the study population The continuous variables were expressed as median and IQR. The categorical variables were expressed as frequency and percentage. IQR: interquartile range; GCS: Glasgow Coma Scale

Parameters	Value
Total participants	425
Age (years)	58.9 (51.7-72.3)
Female	170 (40%)
GCS score at admission	7.0 (4.0-9.0)
Reasons for admission	
Road traffic accidents	137 (32.2%)
Household fall	86 (20.2%)
Stroke (or thromboischemic attacks)	202 (47.5%)
Systolic blood pressure at admission (mmHg)	132.0 (116.0-161.0)
Serum sodium at admission (mEq/L)	137.0 (131.0-141.0)
Comorbidities	
Type 2 diabetes mellitus	310 (72.9%)
Hypertension	275 (64.7%)
Bacterial isolates	
Enterobacterales	253 (59.5%)
Escherichia coli	46 (10.8%)
Klebsiella pneumoniae	162 (38.1%)
*Enterobacter cloacae *complex	12 (2.8%)
Proteus mirabilis	21 (4.9%)
Morganella morganii	1 (0.2%)
Providencia stuartii	2 (0.5%)
Salmonella typhi	2 (0.5%)
Serratia marcescens	7 (1.6%)
Non-fermenters	130 (30.6%)
Acinetobacter baumannii	71 (16.7%)
Pseudomonas aeruginosa	50 (11.8%)
Burkholderia cepacia	7 (1.6%)
Elizabethkingia meningoseptica	2 (0.5%)
Gram-positive cocci	42 (9.9%)
Staphylococcus aureus	32 (7.5%)
Enterococcus faecalis	8 (1.9%)
Enterococcus faecium	2 (0.5%)

**Figure 1:**
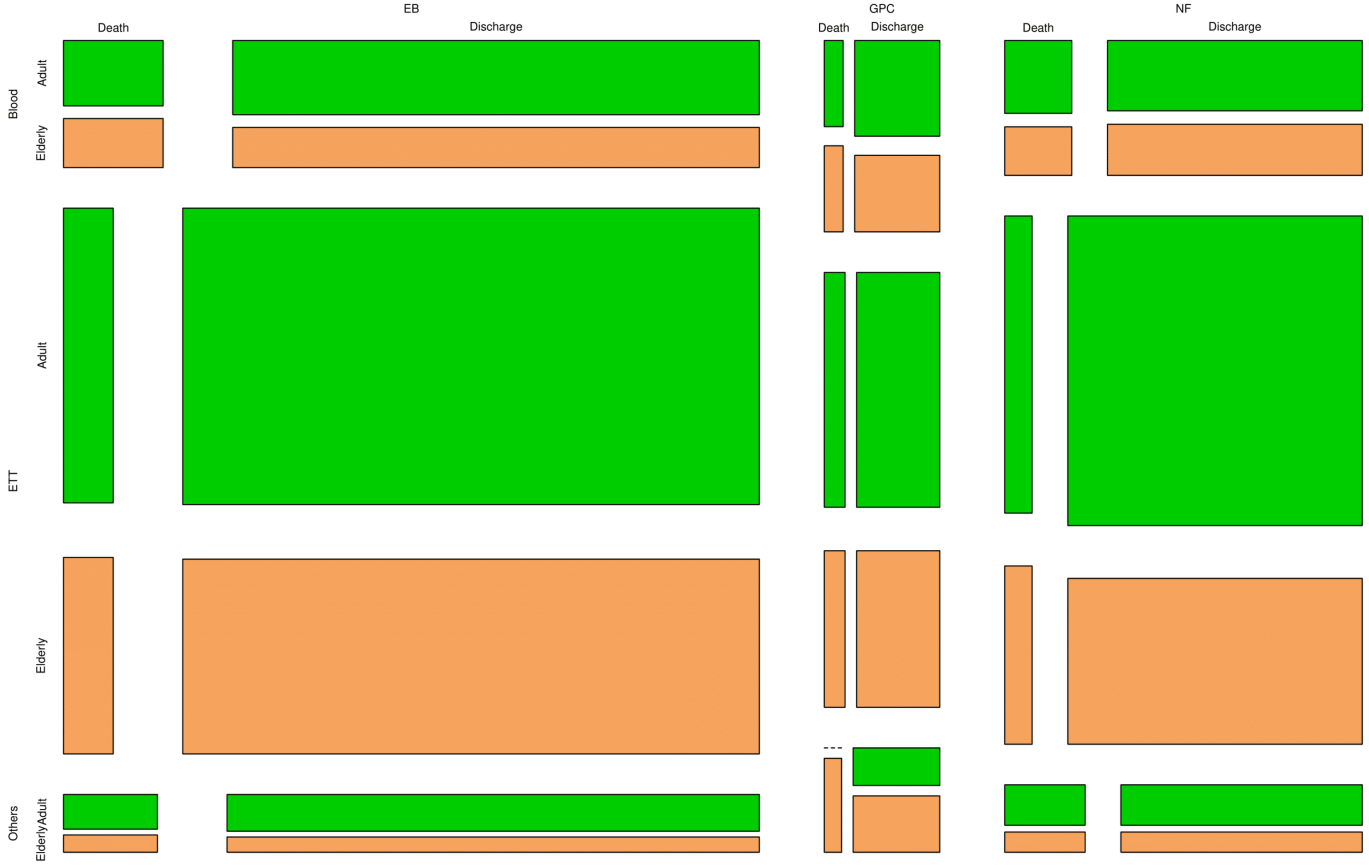
Distribution of the study participants The mosaic plot portrays the distribution of participants per the pathogens detected (EB, GPC, or NF), sample (blood, ETT, or others), age group (adult or elderly), and outcome (discharge or death). EB: *Enterobacterales;* NF: non-fermenters; GPC: Gram-positive cocci; ETT: endotracheal tube This image is an original creation of Jyoti Prakash Sahoo with the use of R software.

Figure 1 portrays the distribution of the study population through a mosaic plot. For grouping, we used the following parameters: pathogen (*Enterobacterales*, Gram-positive cocci, or non-fermenters), sample (blood, ETT, or others), age group (adult or elderly), and outcome (discharge or death). The plot was first grouped by the pathogens detected. The next three divisions were based on sample, outcome, and age group. The majority of samples were obtained from ETT (308, 72%), followed by blood (79, 19%) and others like urine, pus, etc. (38, 9%), respectively. Most of the pathogens belonged to *Enterobacterales* (253, 60%), followed by non-fermenters (130, 30%) and Gram-positive cocci (42, 10%), respectively. Adult persons (262, 62%) outnumbered elderly individuals (163, 38%). Three hundred seventy-six (88.5%) patients were discharged. The remaining 49 (11.5%) patients succumbed to death.

**Figure 2:**
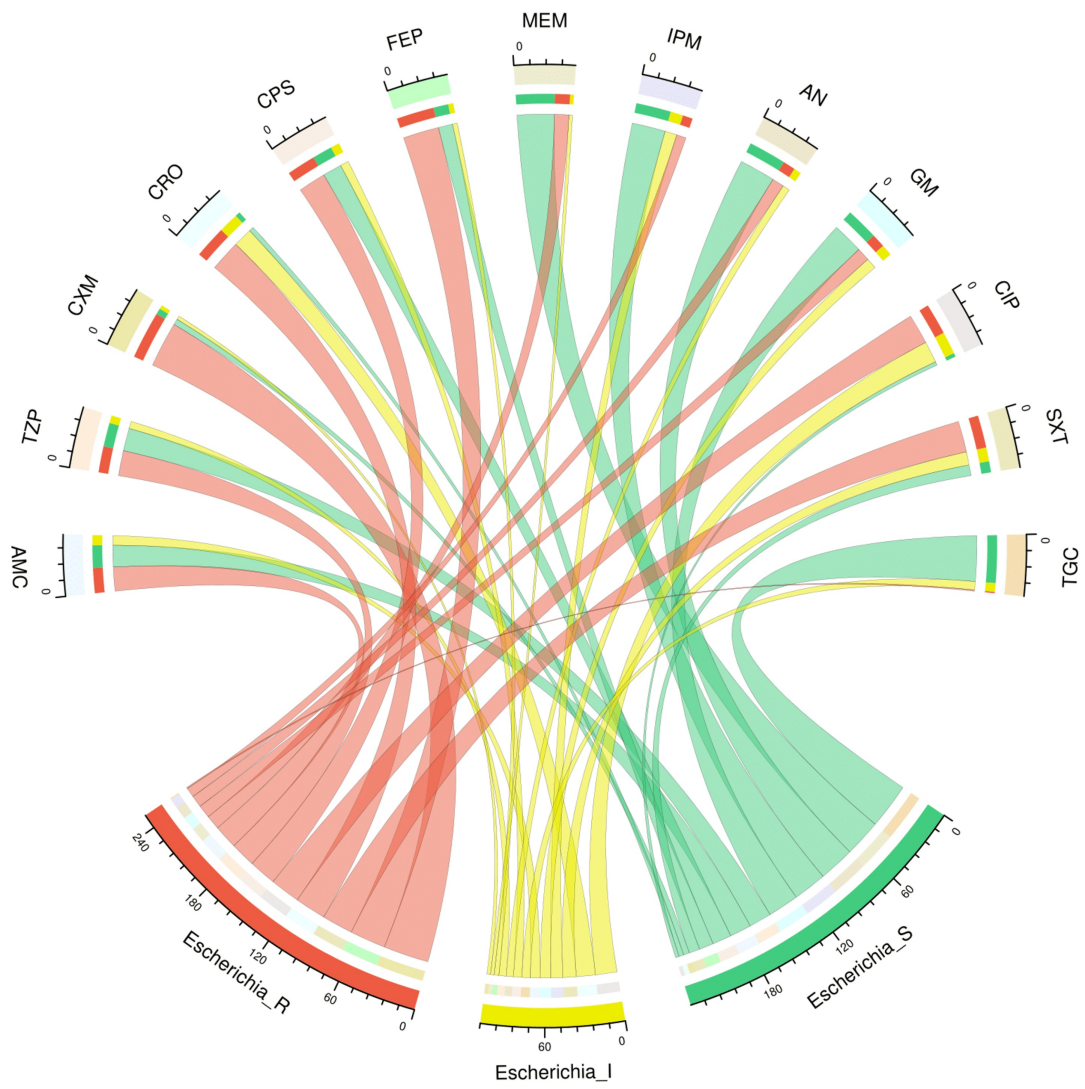
Antibiotic susceptibility pattern of Escherichia coli The upper and lower portions of the chord diagram illustrate the 13 drugs used in AST and the antibiotic susceptibility patterns (R: resistant; I: intermediate; and S: sensitive) of *Escherichia coli*. The bandwidths between the connecting bands of the upper and lower portions correlate with the number of isolates showing sensitivity patterns to various drugs in *Escherichia coli* isolates. AST: antimicrobial susceptibility testing; AMC: amoxicillin-clavulanic acid; TZP: piperacillin-tazobactam; CXM: cefuroxime; CRO: ceftriaxone; CPS: cefoperazone-sulbactam; FEP: cefepime; MEM: meropenem; IPM: imipenem; AN: amikacin; GM: gentamicin; CIP: ciprofloxacin; SXT: cotrimoxazole; TGC: tigecycline This image is an original creation of Jyoti Prakash Sahoo with the use of R software.

**Figure 3:**
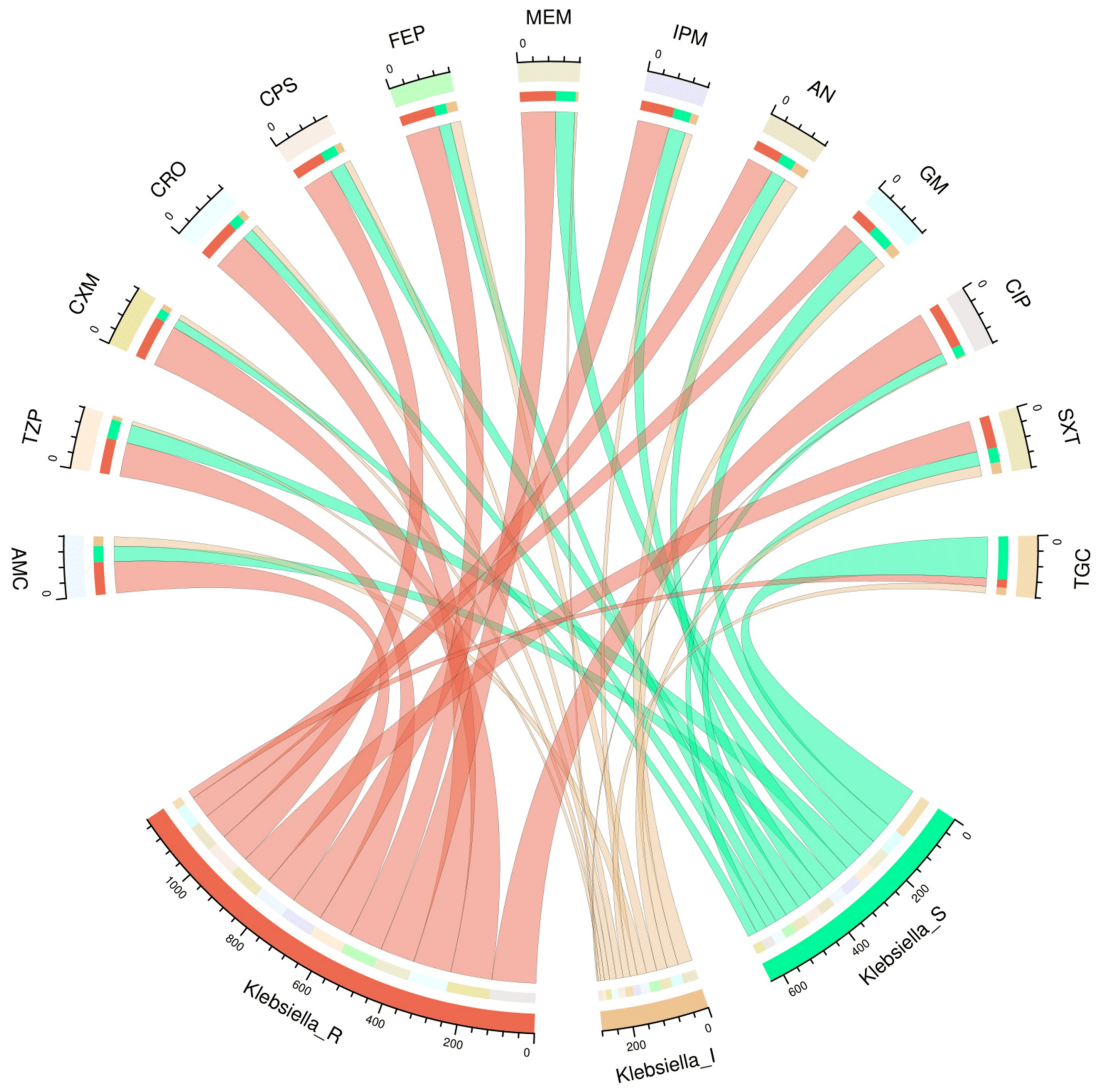
Antibiotic susceptibility pattern of Klebsiella pneumoniae The upper and lower portions of the chord diagram illustrate the 13 drugs used in AST and the antibiotic susceptibility patterns (R: resistant; I: intermediate; and S: sensitive) of *Klebsiella pneumoniae*. The bandwidths between the connecting bands of the upper and lower portions correlate with the number of isolates showing sensitivity patterns to various drugs in *Klebsiella pneumoniae* isolates. AST: antimicrobial susceptibility testing; AMC: amoxicillin-clavulanic acid; TZP: piperacillin-tazobactam; CXM: cefuroxime; CRO: ceftriaxone; CPS: cefoperazone-sulbactam; FEP: cefepime; MEM: meropenem; IPM: imipenem; AN: amikacin; GM: gentamicin; CIP: ciprofloxacin; SXT: cotrimoxazole; TGC: tigecycline This image is an original creation of Jyoti Prakash Sahoo with the use of R software.

Figure 2 and Figure 3 illustrate the antimicrobial susceptibility profiles of 46 isolates of *Escherichia coli* and 162 isolates of *Klebsiella pneumoniae* through chord diagrams, respectively. The *Escherichia coli* isolates demonstrated the highest sensitivity to tigecycline (38, 83%), followed by meropenem (31, 68%), amikacin (30, 65%), and gentamicin (26, 56%). The resistance was maximum against cefuroxime (38, 83%), followed by cefepime (30, 65%), ceftriaxone (26, 56%), and cotrimoxazole (26, 56%). The *Klebsiella pneumoniae* isolates demonstrated the highest sensitivity to tigecycline (119, 74%), followed by gentamicin (74, 40%), amikacin (16, 18%), meropenem (55, 34%), piperacillin-tazobactam (53, 33%), and imipenem (49, 30%). Drug resistance was seen in >50% of isolates against all antimicrobials except tigecycline (23, 14%). Table 2 showcases the numbers and percentages of the antimicrobial susceptibility profiles of all *Enterobacterales* isolates detected in our study.

**Table 2:** Antimicrobial susceptibility test findings of Enterobacterales Antibiotic susceptibility patterns (i.e., sensitive, intermediate, and resistant) of *Enterobacterales* are expressed as frequency and proportions. AMC: amoxicillin-clavulanic acid; TZP: piperacillin-tazobactam; CXM: cefuroxime; CRO: ceftriaxone; CPS: cefoperazone-sulbactam; FEP: cefepime; MEM: meropenem; IPM: imipenem; AN: amikacin; GM: gentamicin; CIP: ciprofloxacin; SXT: cotrimoxazole; TGC: tigecycline

Bacteria	AMC	TZP	CXM	CRO	CPS	FEP	MEM	IPM	AN	GM	CIP	SXT	TGC
*Escherichia coli* (n=46)													
Sensitive	18 (39%)	19 (41%)	5 (11%)	4 (9%)	16 (35%)	12 (26%)	31 (68%)	28 (61%)	30 (65%)	26 (56%)	3 (6%)	9 (20%)	38 (83%)
Intermediate	8 (17%)	6 (13%)	3 (6%)	16 (35%)	7 (15%)	4 (9%)	3 (6%)	10 (22%)	6 (13%)	9 (20%)	18 (39%)	11 (24%)	7 (15%)
Resistant	20 (44%)	21 (46%)	38 (83%)	26 (56%)	23 (50%)	30 (65%)	12 (26%)	8 (17%)	10 (22%)	11 (24%)	25 (55%)	26 (56%)	1 (2%)
*Klebsiella pneumoniae* (n=162)													
Sensitive	44 (27%)	53 (33%)	27 (17%)	33 (20%)	46 (28%)	34 (21%)	55 (34%)	49 (30%)	43 (26%)	64 (40%)	31 (19%)	41 (25%)	119 (74%)
Intermediate	27 (17%)	11 (7%)	15 (9%)	19 (12%)	23 (14%)	30 (19%)	7 (4%)	21 (13%)	42 (26%)	33 (20%)	4 (2%)	31 (19%)	20 (12%)
Resistant	91 (56%)	98 (60%)	120 (74%)	110 (68%)	93 (58%)	98 (60%)	100 (62%)	92 (57%)	77 (48%)	65 (40%)	127 (79%)	90 (56%)	23 (14%)
*Enterobacter cloacae* complex (n=12)													
Sensitive	0	7 (59%)	0	6 (50%)	8 (66%)	8 (66%)	10 (83%)	7 (59%)	10 (83%)	11 (92%)	8 (66%)	11 (92%)	11 (92%)
Intermediate	1 (8%)	1 (8%)	1 (8%)	0	2 (17%)	0	0	2 (17%)	0	0	2 (17%)	0	0
Resistant	11 (92%)	4 (33%)	11 (92%)	6 (50%)	2 (17%)	4 (34%)	2 (17%)	3 (24%)	2 (17%)	1 (8%)	2 (17%)	1 (8%)	1 (8%)
*Proteus mirabilis* (n=21)													
Sensitive	0	14 (67%)	0	0	0	3 (14%)	3 (14%)	0	0	4 (19%)	0	7 (33%)	6 (29%)
Intermediate	8 (38%)	4 (19%)	2 (10%)	3 (14%)	1 (5%)	0	1 (5%)	1 (5%)	0	3 (14%)	0	0	3 (14%)
Resistant	13 (62%)	3 (14%)	19 (90%)	18 (86%)	20 (95%)	18 (86%)	17 (81%)	20 (95%)	21 (100%)	14 (67%)	21 (100%)	14 (67%)	12 (57%)
*Morganella morganii* (n=1)													
Sensitive	0	1 (100%)	0	1 (100%)	1 (100%)	0	1 (100%)	0	1 (100%)	1 (100%)	0	0	0
Intermediate	0	0	0	0	0	0	0	0	0	0	0	0	0
Resistant	1 (100%)	0	1 (100%)	0	0	0	0	1 (100%)	0	0	1 (100%)	1 (100%)	1 (100%)
*Providencia stuartii* (n=2)													
Sensitive	0	2 (100%)	0	0	0	0	1 (50%)	0	0	0	1 (50%)	1 (50%)	1 (50%)
Intermediate	0	0	0	0	1 (50%)	0	0	0	0	0	0	0	0
Resistant	2 (100%)	0	2 (100%)	2 (100%)	1 (50%)	2 (100%)	1 (50%)	2 (100%)	2 (100%)	2 (100%)	1 (50%)	1 (50%)	1 (50%)
*Salmonella typhi* (n=2)													
Sensitive	2 (100%)	0	2 (100%)	0	0	0	0	0	2 (100%)	2 (100%)	2 (100%)	0	0
Intermediate	0	0	0	1 (50%)	1 (50%)	1 (50%)	0	0	0	0	0	0	0
Resistant	0	2 (100%)	0	1 (50%)	1 (50%)	1 (50%)	2 (100%)	2 (100%)	0	0	0	2 (100%)	2 (100%)
*Serratia marcescens* (n=7)													
Sensitive	2 (29%)	4 (57%)	2 (29%)	1 (14%)	4 (57%)	2 (29%)	2 (29%)	2 (29%)	2 (29%)	2 (29%)	2 (29%)	2 (29%)	2 (29%)
Intermediate	2 (29%)	1 (14%)	0	2 (29%)	2 (29%)	0	1 (14%)	2 (29%)	0	0	0	2 (29%)	2 (29%)
Resistant	3 (42%)	2 (29%)	5 (71%)	4 (57%)	1 (14%)	5 (71%)	4 (57%)	3 (42%)	5 (71%)	5 (71%)	5 (71%)	3 (42%)	3 (42%)

**Figure 4:**
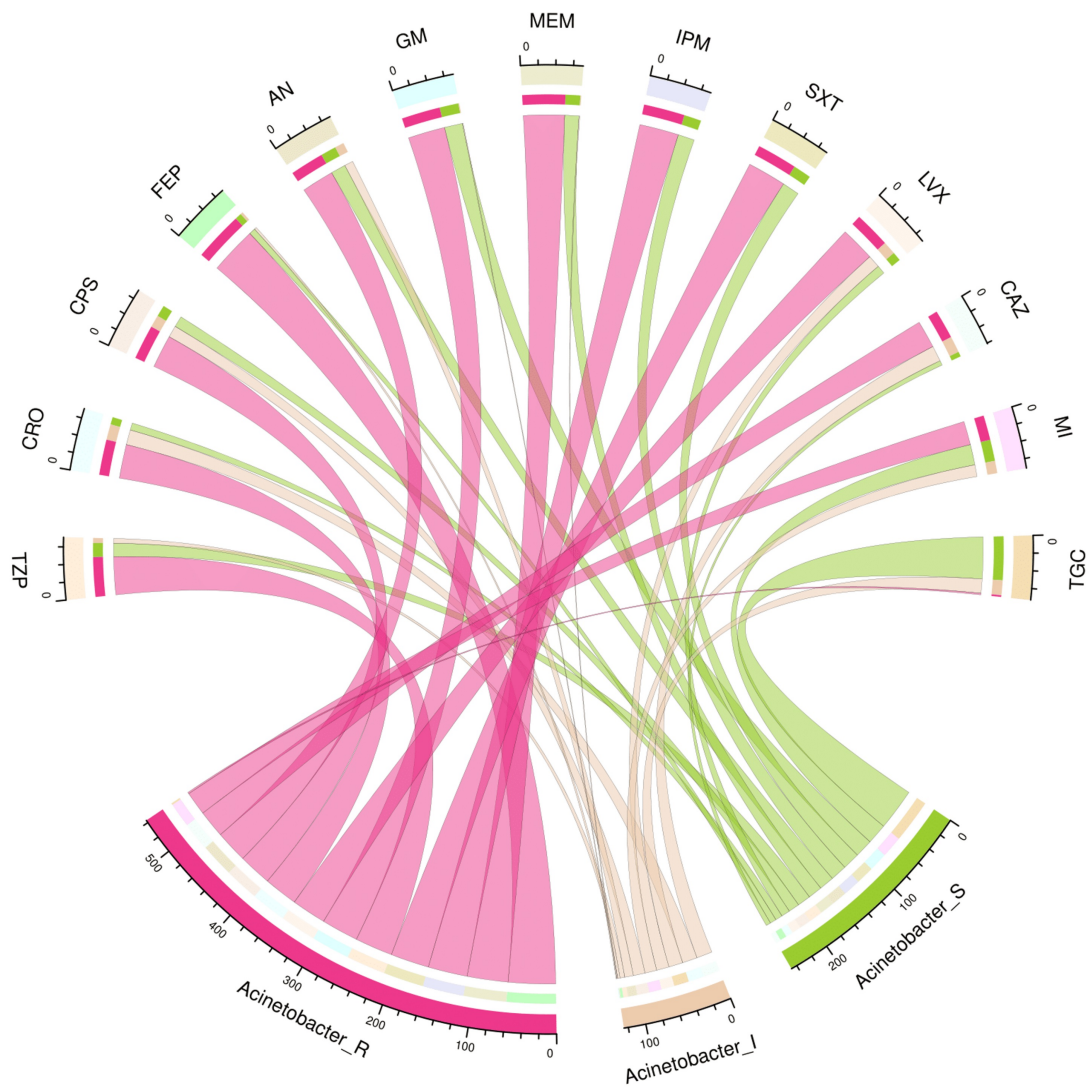
Antibiotic susceptibility pattern of Acinetobacter baumannii The upper and lower portions of the chord diagram illustrate the 13 drugs used in AST and the antibiotic susceptibility patterns (R: resistant; I: intermediate; and S: sensitive) of *Acinetobacter baumannii*. The bandwidths between the connecting bands of the upper and lower portions correlate with the number of isolates showing sensitivity patterns to various drugs in *Acinetobacter baumannii* isolates. AST: antimicrobial susceptibility testing; TZP: piperacillin-tazobactam; CRO: ceftriaxone; CPS: cefoperazone-sulbactam; FEP: cefepime; AN: amikacin; GM: gentamicin; MEM: meropenem; IPM: imipenem; SXT: cotrimoxazole; LVX: levofloxacin; CAZ: ceftazidime; MI: minocycline; TGC: tigecycline This image is an original creation of Jyoti Prakash Sahoo with the use of R software.

**Figure 5:**
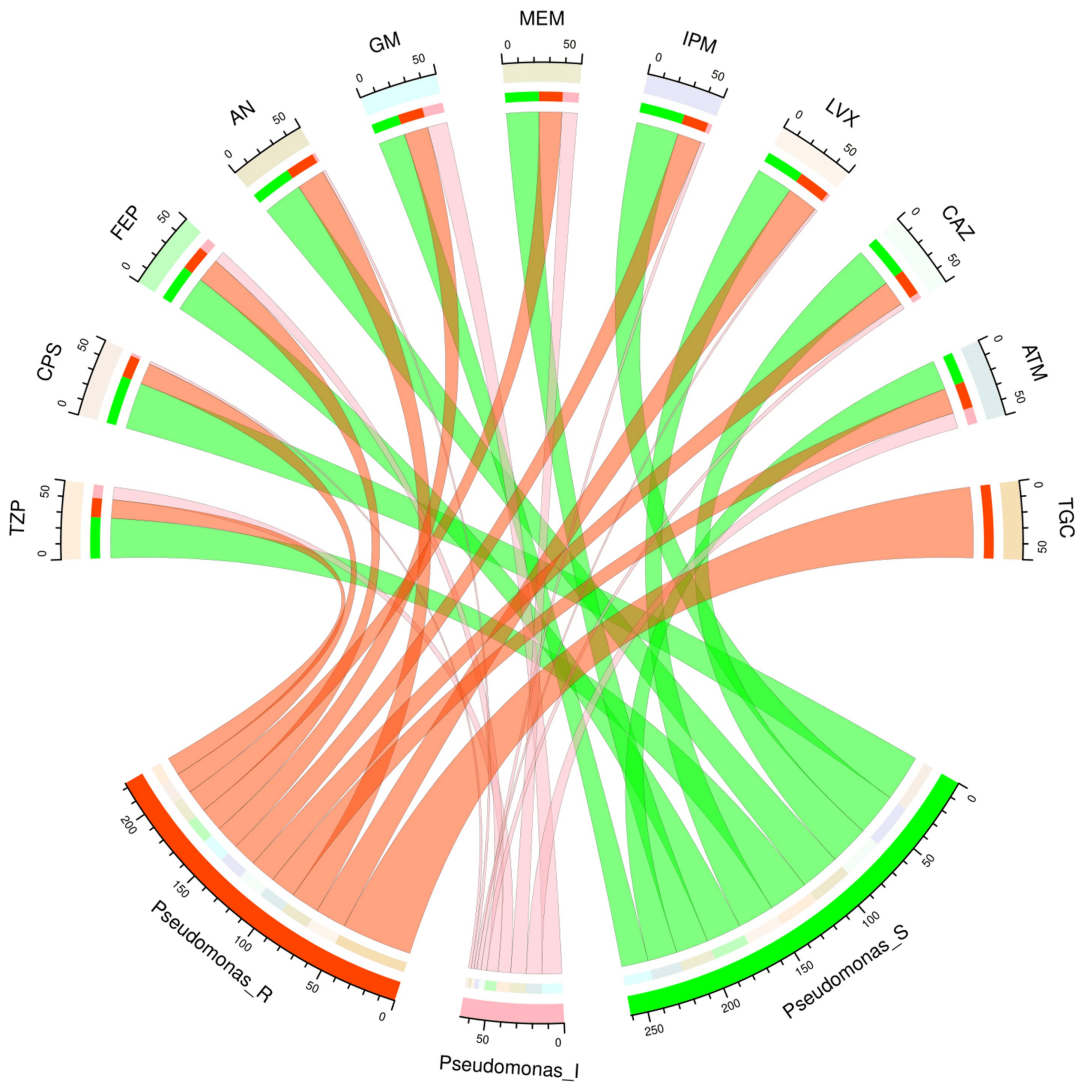
Antibiotic susceptibility pattern of Pseudomonas aeruginosa The upper and lower portions of the chord diagram illustrate the 11 drugs used in AST and the antibiotic susceptibility patterns (R: resistant; I: intermediate; and S: sensitive) of *Pseudomonas aeruginosa*. The bandwidths between the connecting bands of the upper and lower portions correlate with the number of isolates showing sensitivity patterns to various drugs in *Pseudomonas aeruginosa* isolates. AST: antimicrobial susceptibility testing; TZP: piperacillin-tazobactam; CPS: cefoperazone-sulbactam; FEP: cefepime; AN: amikacin; GM: gentamicin; MEM: meropenem; IPM: imipenem; LVX: levofloxacin; CAZ: ceftazidime; ATM: aztreonam; TGC: tigecycline This image is an original creation of Jyoti Prakash Sahoo with the use of R software.

Figure 4 and Figure 5 illustrate the antimicrobial susceptibility profiles of 71 isolates of *Acinetobacter baumannii* and 50 isolates of *Pseudomonas aeruginosa* through chord diagrams, respectively. The *Acinetobacter baumannii* isolates demonstrated the highest sensitivity to tigecycline (51, 72%), followed by minocycline (26, 37%), gentamicin (23, 32%), cotrimoxazole (22, 31%), and imipenem (21, 30%). The highest and lowest resistance cases were observed against cefepime (60, 85%) and tigecycline (2, 3%). The *Pseudomonas aeruginosa* isolates demonstrated the highest sensitivity to cefoperazone-sulbactam (33, 66%), followed by imipenem (30, 60%), piperacillin-tazobactam (28, 56%), amikacin (28, 56%), and ceftazidime (28, 56%). All 50 isolates demonstrated resistance against tigecycline. Among the other drugs, resistance was the maximum against amikacin (20, 40%), ceftazidime (18, 36%), and aztreonam (18, 36%). Table 3 showcases the numbers and percentages of the antimicrobial susceptibility profiles of all non-fermenter isolates detected in our study.

**Table 3:** Antimicrobial susceptibility test findings of non-fermenters Antibiotic susceptibility patterns (i.e., sensitive, intermediate, and resistant) of non-fermenters are expressed as frequency and proportions. TZP: piperacillin-tazobactam; CRO: ceftriaxone; CPS: cefoperazone-sulbactam; FEP: cefepime; AN: amikacin; GM: gentamicin; MEM: meropenem; IPM: imipenem; SXT: cotrimoxazole; LVX: levofloxacin; CAZ: ceftazidime; MI: minocycline; ATM: aztreonam; TGC: tigecycline; NA: not applicable

Bacteria	TZP	CRO	CPS	FEP	AN	GM	MEM	IPM	SXT	LVX	CAZ	MI	ATM	TGC
*Acinetobacter baumannii* (n=71)														
Sensitive	17 (24%)	9 (13%)	14 (20%)	8 (11%)	19 (27%)	23 (32%)	18 (25%)	21 (30%)	22 (31%)	11 (15%)	9 (13%)	26 (37%)	NA	51 (72%)
Intermediate	6 (8%)	19 (27%)	15 (21%)	3 (4%)	11 (15%)	1 (2%)	1 (2%)	0	0	16 (23%)	23 (32%)	15 (21%)		18 (25%)
Resistant	48 (68%)	43 (60%)	42 (59%)	60 (85%)	41 (58%)	47 (66%)	52 (73%)	50 (70%)	49 (69%)	44 (62%)	39 (55%)	30 (42%)		2 (3%)
*Pseudomonas aeruginosa* (n=50)														
Sensitive	28 (56%)	NA	33 (66%)	25 (50%)	28 (56%)	19 (38%)	23 (46%)	30 (60%)	NA	26 (52%)	28 (56%)	NA	21 (42%)	0
Intermediate	9 (18%)		2 (4%)	8 (16%)	2 (4%)	14 (28%)	11 (22%)	3 (6%)		2 (4%)	4 (8%)		11 (22%)	0
Resistant	13 (26%)		15 (30%)	17 (34%)	20 (40%)	17 (34%)	16 (32%)	17 (34%)		22 (44%)	18 (36%)		18 (36%)	50 (100%)
*Burkholderia cepacia* (n=7)														
Sensitive	0	0	4 (57%)	0	0	0	5 (71%)	0	5 (71%)	5 (71%)	4 (57%)	6 (86%)	0	0
Intermediate	1 (14%)	0	0	1 (14%)	2 (29%)	1 (14%)	0	0	0	0	0	0	1 (14%)	2 (29%)
Resistant	6 (86%)	7 (100%)	3 (43%)	6 (86%)	5 (71%)	6 (86%)	2 (29%)	7 (32%)	2 (29%)	2 (29%)	3 (43%)	1 (14%)	6 (86%)	5 (71%)
*Elizabethkingia meningoseptica* (n=2)														
Sensitive	0	0	0	0	0	0	0	0	0	0	0	1 (50%)	0	NA
Intermediate	0	1 (50%)	0	0	0	1 (50%)	0	0	0	1 (50%)	1 (50%)	0	1 (50%)	
Resistant	2 (100%)	1 (50%)	2 (100%)	2 (100%)	2 (100%)	1 (50%)	2 (100%)	2 (100%)	2 (100%)	1 (50%)	1 (50%)	1 (50%)	1 (50%)	

**Figure 6:**
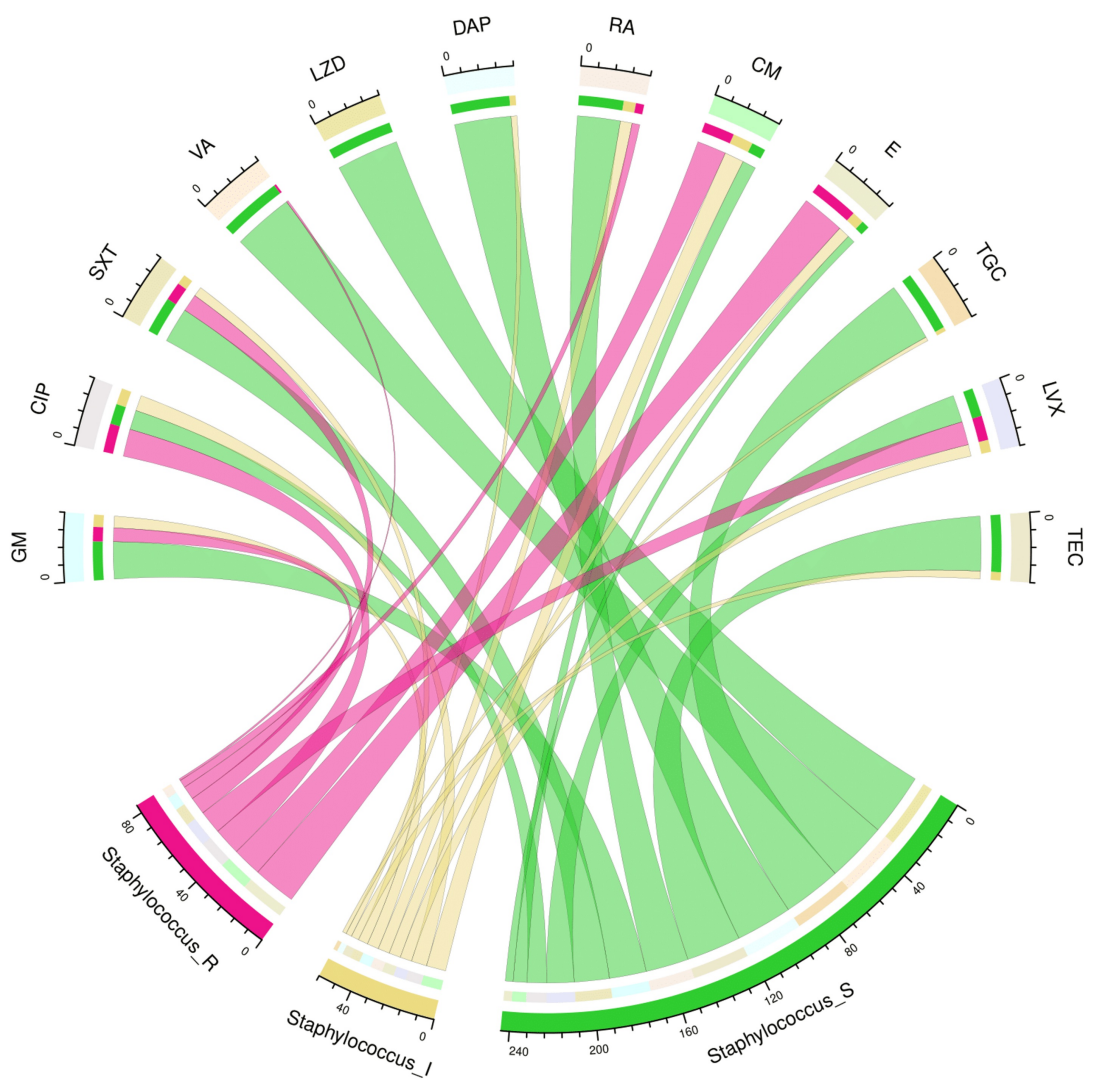
Antibiotic susceptibility pattern of Staphylococcus aureus The upper and lower portions of the chord diagram illustrate the 12 drugs used in AST and the antibiotic susceptibility patterns (R: resistant; I: intermediate; and S: sensitive) of *Staphylococcus aureus.* The bandwidths between the connecting bands of the upper and lower portions correlate with the number of isolates showing sensitivity patterns to various drugs in *Staphylococcus aureus* isolates. AST: antimicrobial susceptibility testing; GM: gentamicin; CIP: ciprofloxacin; SXT: cotrimoxazole; VA: vancomycin; LZD: linezolid; DAP: daptomycin; RA: rifampicin; CM: clindamycin; E: erythromycin; TGC: tigecycline; LVX: levofloxacin; TEC: teicoplanin This image is an original creation of Jyoti Prakash Sahoo with the use of R software.

Figure 6 illustrates the antimicrobial susceptibility profile of 32 *Staphylococcus aureus* isolates through a chord diagram. The *Staphylococcus aureus* isolates demonstrated the highest sensitivity to linezolid (32, 100%), followed by vancomycin (31, 97%), tigecycline (30, 94%), daptomycin (29, 91%), and teicoplanin (28, 88%). The resistance was maximum against erythromycin (22, 69%), followed by clindamycin (15, 47%), ciprofloxacin (14, 44%), and levofloxacin (12, 37%). Table 4 showcases the numbers and percentages of the antimicrobial susceptibility profiles of all Gram-positive cocci detected in our study.

**Table 4:** Antimicrobial susceptibility test findings of Gram-positive cocci The antibiotic susceptibility patterns (i.e., sensitive, intermediate, and resistant) of Gram-positive cocci are expressed as frequency and proportions. GM: gentamicin; CIP: ciprofloxacin; SXT: cotrimoxazole; VA: vancomycin; LZD: linezolid; DAP: daptomycin; RA: rifampicin; CM: clindamycin; E: erythromycin; TGC: tigecycline; LVX: levofloxacin; TEC: teicoplanin

Bacteria	GM	CIP	SXT	VA	LZD	DAP	RA	CM	E	TGC	LVX	TEC
*Staphylococcus aureus* (n=32)												
Sensitive	19 (59%)	10 (31%)	18 (56%)	31 (97%)	32 (100%)	29 (91%)	22 (69%)	7 (22%)	4 (12%)	30 (94%)	14 (44%)	28 (88%)
Intermediate	6 (19%)	8 (25%)	5 (16%)	0	0	3 (9%)	6 (19%)	10 (31%)	6 (19%)	2 (6%)	6 (19%)	4 (12%)
Resistant	7 (22%)	14 (44%)	9 (28%)	1 (3%)	0	0	4 (12%)	15 (47%)	22 (69%)	0	12 (37%)	0
*Enterococcus faecalis* (n=8)												
Sensitive	0	2 (25%)	0	8 (100%)	8 (100%)	6 (75%)	0	0	0	8 (100%)	4 (50%)	8 (100%)
Intermediate	0	0	0	0	0	2 (25%)	0	0	3 (37%)	0	2 (25%)	0
Resistant	8 (100%)	6 (75%)	8 (100%)	0	0	0	8 (100%)	8 (100%)	5 (63%)	0	2 (25%)	0
*Enterococcus faecium* (n=2)												
Sensitive	0	0	0	2 (100%)	2 (100%)	2 (100%)	0	0	0	2 (100%)	0	2 (100%)
Intermediate	0	0	0	0	0	0	0	0	0	0	1 (50%)	0
Resistant	2 (100%)	2 (100%)	2 (100%)	0	0	0	2 (100%)	2 (100%)	2 (100%)	0	1 (50%)	0

## Discussion

Four hundred twenty-five (26.7%) of the 1589 patients admitted to the neurosurgery ICU were assessed for bacteriological profiles and AST patterns. Two hundred fifty-five males totalled 60% of the participants. The median age of the participants was 58.9 (51.7-72.3) years. The median GCS score was 7.0 (4.0-9.0). *Klebsiella pneumoniae* (162, 38.1%) and *Escherichia coli* (46, 10.8%) were the *Enterobacterales* most commonly found. *Staphylococcus aureus* (32, 7.5%) was the most prevalent Gram-positive coccus in our sample, while *Acinetobacter baumannii* (71, 16.7%) and *Pseudomonas aeruginosa* (50, 11.8%) were the most common non-fermenters. Similar bacteriological profiles and AST patterns had been found in our earlier paper [26]. These findings are consistent with prior studies in ICU populations [17,18], but our study adds neurosurgery-specific data from an Indian tertiary care setting.

The majority of our participants had hypertension and/or type 2 diabetes. People are admitted to the neurosurgery ICU nowadays due to cerebrovascular accidents, falls in the home, or traffic accidents [27]. The majority of patients admitted following traffic accidents were younger men. Male elderly patients with hypertension are more likely to experience a stroke or transient ischemic attack [12,19,26,27]. Longer hospital stays, decreased mobility, slower mucociliary clearing of airways, and impaired consciousness contribute to hospital-acquired infections (HAI) [27-29]. Antimicrobial resistance is more common due to irrational antibiotic use [26,28]. Of the 425 bacterial isolates, *Klebsiella pneumoniae*, *Acinetobacter baumannii*, *Pseudomonas aeruginosa*, *Escherichia coli*, and *Staphylococcus aureus* were the most frequently observed pathogens. Additionally, we spotted emergent pathogenic bacteria such as *Proteus mirabilis*, *Serratia marcescens*, *Morganella morganii*, and *Elizabethkingia meningoseptica*. Resistance in ICU pathogens is also driven by biofilm formation, quorum sensing, and device-associated colonization [27-29].

*Klebsiella pneumoniae* and *Escherichia coli* isolates were most sensitive to tigecycline. *Proteus mirabilis* and *Serratia marcescens* isolates were mainly sensitive to piperacillin-tazobactam. These patterns concorded with our previous study [26]. *Acinetobacter baumannii* isolates showed the highest sensitivity to tigecycline and minocycline. *Pseudomonas aeruginosa* isolates showed the highest sensitivity to cefoperazone-sulbactam and imipenem. The non-fermenters' AST pattern portrayed a new sensitivity trend compared to our previous study [26]. In that study, we found that minocycline and imipenem had the highest efficacy against *Acinetobacter baumannii* and *Pseudomonas aeruginosa*. *Elizabethkingia meningoseptica* was found to be sensitive to minocycline and colistin in a recent investigation by Wu et al. [30]. The only effective medication in our study against *Elizabethkingia meningoseptica* was minocycline. Our previous study [26] also supported this finding. Linezolid was effective against all *Staphylococcus aureus* isolates. Their sensitivities towards vancomycin, tigecycline, daptomycin, and teicoplanin were very good. These five antimicrobials were also very effective against *Enterococcus* species.

The study's primary strength was the data visualization through mosaic plots and chord diagrams. Our study also had a few limitations. First, the comorbidities and concurrent drugs were not investigated. Second, we did not look at the relationship between the outcome, duration of hospital stays, and AST results. Third, there are several risk factors and etiologies for HAI in ICU patients. No risk factors for the emergence of HAI were evaluated in this study. Fourth, we did not consider the device-day denominators, multidrug-resistant/extensively drug-resistant (MDR/XDR) definitions, comorbidities, and concomitant medications for the analysis of AST patterns. Fifth, there could be some possible VITEK misidentifications of rare species.

## Conclusions

The most common sample collected was ETT, and most pathogens belonged to *Enterobacterales*. *Klebsiella pneumoniae*, *Escherichia coli*, *Acinetobacter baumannii*, *Pseudomonas aeruginosa*, and *Staphylococcus aureus* were the most common pathogenic bacteria encountered. We also found emerging pathogens like *Elizabethkingia meningoseptica* and *Morganella morganii*. *Klebsiella pneumoniae*, *Escherichia coli*, and *Acinetobacter baumannii* demonstrated maximum sensitivity towards tigecycline. Cefoperazone-sulbactam was highly effective against *Pseudomonas aeruginosa*. *Staphylococcus aureus* showed maximum sensitivity towards linezolid, vancomycin, tigecycline, daptomycin, and teicoplanin. We suggest prospective studies with a longer duration to determine the trends of the bacteriological profile and their AST patterns.
